# Reduced folate receptor alpha (FOLR1) protein expression in fallopian tubes from premenopausal women: implications for the FOLR1 CDx assay for mirvetuximab‐soravtansine therapy

**DOI:** 10.1002/2056-4538.70091

**Published:** 2026-04-23

**Authors:** Annika Nasdala, Leonie D Kandt, Martin Radner, Nora Schaumann, Malte Gronewold, Pia Hillmann, Henriette Christgen, Jens Hachenberg, Elna Kuehnle, Christian Hartmann, Matthias Christgen

**Affiliations:** ^1^ Institute of Pathology Hannover Medical School Hannover Germany; ^2^ Department of Obstetrics and Gynecology Hannover Medical School Hannover Germany

**Keywords:** ovarian carcinoma, standardized controls, reference materials, immunohistochemistry, folate receptor alpha (FOLR1)

## Abstract

Mirvetuximab‐soravtansine (MIRV‐S) is an antibody‐drug conjugate targeting folate receptor alpha (FOLR1). MIRV‐S is approved for the treatment of FOLR1‐positive, platinum‐resistant ovarian carcinoma. Patient eligibility is determined by immunohistochemistry (IHC) using a companion diagnostic (CDx) assay (FOLR1‐2.1, Ventana). This assay requires on‐slide positive controls (OPCs) to aid FOLR1 evaluation. The manufacturer recommends normal fallopian tube (NFT) tissue for OPCs. Estrogen receptor signaling represses FOLR1 in cell culture models. It is unknown whether hormonal factors, such as menopausal status, also impact on FOLR1 immunoreactivity in NFTs used as OPCs. To address this question, we studied FOLR1 protein expression in NFTs (n = 51) from women aged 26–83 years. IHC was performed with the FOLR1‐2.1 CDx assay. Immunoreactivity at apical and basolateral cell membranes was assessed using H‐scores (aH‐score and bH‐score respectively). Overall FOLR1 expression was evaluated using a combined H‐score (cH‐score; i.e. aH‐ and bH‐scores added together). Immunoreactivity scores in pre‐, peri‐, and postmenopausal age groups were compared with the chi‐square test for trends. NFTs showed variable FOLR1 protein expression [median aH‐score: 152.5, interquartile range (IQR): 120–175; median bH‐score: 35, IQR: 7–85; median cH‐score: 195, IQR: 140–245]. Apical immunoreactivity was age‐independent (*p* = 0.619), but low or absent basolateral immunoreactivity (bH‐score <35) was associated with premenopausal age (*p* = 0.018). Low overall FOLR1 expression (cH‐score <195) was also associated with premenopausal age (*p* = 0.037). In conclusion, NFTs show an age‐dependent FOLR1 expression pattern, which likely reflects hormonal repression of FOLR1 in premenopausal women. NFT tissue from postmenopausal women is appropriate and meets the requirements for the current FOLR1 CDx assay.

## Introduction

Mirvetuximab‐soravtansine (MIRV‐S) is an antibody‐drug conjugate (ADC) targeting the folate receptor alpha (FOLR1) molecule [1, 2]. FOLR1 is a cell surface glycoprotein that regulates the uptake of folate, a coenzyme required for DNA synthesis. Most normal tissues lack FOLR1 protein expression. In FOLR1‐negative tissues, the RCF carrier facilitates folate uptake [3]. Few specialized cell types express FOLR1. These include: (1) choroid plexus (CP) epithelial cells, (2) kidney epithelial cells, and (3) normal fallopian tube (NFT) epithelial cells [4, 5]. In CP epithelial cells, FOLR1 is involved in the regulation of folate concentrations in the cerebrospinal fluid [6]. In the kidney, FOLR1 functions as a salvage receptor that returns folate to the blood [7, 8]. In NFT tissue, the function of FOLR1 has remained unclear [3]. Ovarian carcinomas express FOLR1 at variable levels, and it is assumed that FOLR1‐dependent folate uptake contributes to enhanced DNA synthesis and cell proliferation in a proportion of these tumors [7, 9].

FOLR1 has been investigated as a target for drug delivery for more than two decades [10]. In November 2022, the Food and Drug Administration (FDA) approved MIRV‐S (trade name Elahere) for the treatment of FOLR1‐positive, platinum‐resistant ovarian carcinoma in the United States [1]. Two years later, in November 2024, the European Medicines Agency (EMA) approved MIRV‐S for the treatment of patients with FOLR1‐positive, platinum‐resistant ovarian carcinoma in the European Union (EU). These decisions were based on two clinical trials (SORAYA trial, MIRASOL trial), which enrolled patients with platinum‐resistant, FOLR1‐positive ovarian carcinoma [1, 11]. The SORAYA trial, a phase 3 single‐arm trial, documented an objective response rate (ORR) of 32.4% [11]. The MIRASOL trial, a phase 3 double‐arm trial, showed that median overall survival was longer with MIRV‐S than with conventional chemotherapy (16.5 months versus 12.8 months) [1]. Both clinical trials utilized the same immunohistochemical assay to determine FOLR1 protein expression and patient eligibility for MIRV‐S therapy (FOLR1‐2.1 assay, Ventana Medical Inc., Tucson, AZ, USA). Using this assay, ovarian carcinomas were classified as FOLR1‐positive if ≥75% of viable tumor cells showed FOLR1 immunoreactivity with at least 2+ staining intensity [1, 11]. FOLR1‐positivity rates in screened patient cohorts were 29% (106 out of 370) in the SORAYA trial and 32% (737 out of 2,307) in the MIRASOL trial [1, 11]. The FOLR1‐2.1 assay received FDA approval as a companion diagnostic (CDx) test for MIRV‐S therapy [12]. In the United States, patient selection for MIRV‐S therapy is tied to the FOLR1‐2.1 assay. In the EU, immunohistochemical FOLR1 assessment with alternative anti‐FOLR1 antibodies is theoretically possible if assays comply with the requirements of the EU's *in vitro* diagnostic regulation (IVDR). However, the vast majority of institutions use the FOLR1‐2.1 assay.

External on‐slide positive controls (OPCs) and negative controls (ONCs) have become a mandatory quality assurance measure for diagnostic immunohistochemistry (IHC) [13, 14, 15, 16]. OPCs and ONCs serve multiple critical functions, including monitoring analytical sensitivity and demonstrating the achievement of low limit of detection (LLOD) [13, 14]. In the most severe scenarios, OPCs and ONCs identify false‐negative or false‐positive IHC stains and thus prevent reporting of incorrect test results. OPCs and ONCs are prepared in‐house by histopathology institutions, using anonymized, leftover archival tissues [13, 14]. The Medical Devices Law (MDL/MPG) authenticates the corresponding standard operating procedures (SOP) for OPC/ONC preparation in the European Union (EU).

Significant efforts have been undertaken to improve standardization of IHC assays in the field of diagnostic pathology [13, 14, 15, 16]. Ultimately, this also requires standardization of IHC controls (OPCs and ONCs) [14]. In this context, expert committees have developed the immunohistochemistry critical assay performance controls (iCAPCs) concept [14]. It was proposed that iCAPCs are human positive control tissues with evidence‐based knowledge about their predictable levels and patterns of expression of specified antigens under defined conditions [14]. For instance, appendix vermiformis is a validated iCAPCs for CK20 IHC [14, 17]. It was envisioned that iCAPCs may come to represent the ‘gold standard’ for OPCs, especially for predictive markers or class II assays, where test results direct patient treatment [14]. For class II assays, it is recommended to assemble several iCAPCs in a single multi‐tissue block that incorporates weak, strong and negative expressors [14]. ‘Weak expressor’ iCAPCs are particularly important. They confirm an appropriately LLOD [14]. Newly built multi‐tissue blocks harboring newly sampled iCAPCs are subjected to lot‐to‐lot validation before clinical use as OPCs [14].

The development and validation of reference materials for IHC is an emerging field, which has made progress in recent years. Glass microbeads coated with defined amounts of synthetic peptides have become available for some antigens, including ER, PR, HER2, and PD‐L1 [18, 19, 20, 21, 22, 23, 24]. For the first time, these synthetic calibrators have facilitated the exact evaluation of IHC performance across a range of precisely defined analyte‐concentrations and among different antibody clones. Using this technique, Sompuram *et al* demonstrated that different anti‐PD‐L1 antibody clones possess different LLOD thresholds, which has been suspected by pathologists for many years [20].

Concerning the FOLR1‐2.1 CDx assay, the manufacturer recommends that normal fallopian tube (NFT) tissue should be employed as a single, combined external ONC/OPC [12]. According to the manufacturer's instructions, NFT epithelial cells provide a reference for strong staining intensity (3+) at apical cell membranes, a reference for moderate staining intensity (2+) at basolateral cell membranes, and a reference for absent staining (0) in the mesenchymal stroma [12]. Our laboratory at the Hannover Medical School (MHH, Hannover, Germany) introduced the FOLR1‐2.1 assay in December 2024. Consistent with our general laboratory SOPs, we performed lot‐to‐lot validations for newly prepared NFT specimens. In early 2025, lot‐to‐lot‐validation showed that some NFTs displayed only weak staining (1+) or absent staining (0) at basolateral membranes. The corresponding NFTs were excluded from OPC use. However, this complicated maintenance of the FOLR1 assay and delayed clinical tests. Having experienced these OPC issues, we hypothesized that FOLR1 expression levels in NFTs actually differ between individuals. This hypothesis was reasonable for four reasons: (1) estrogen receptor (ER)‐signaling represses the *FOLR1* gene in various cell culture models [25]; (2) ER binds directly to the *FOLR1* gene promoter, and pharmacological ER antagonists and ER modifiers (tamoxifen and fulvestrant) induce an upregulation of FOLR1 protein expression in IGROV‐1 and BG‐1 ovarian carcinoma cells *in vitro* [25]; (3) NFT epithelial cells are ER‐positive and are estrogen‐responsive [26]; (4) our external OPCs had been prepared from NFTs of elderly (postmenopausal) women or NFTs of young (premenopausal) women, some of whom had just undergone cesarean section with synchronous tubal sterilization. Of note, serum estrogen (E_2_) concentrations are consistently high during the second and third trimester of pregnancy (>500‐fold higher than in postmenopausal women) [27].

Repression of FOLR1 by ER‐signaling raises the possibility that hormonal factors, such as the menopausal status, influence FOLR1 immunoreactivity in NFTs. To address this question and to understand which NFTs typically meet or miss the OPC performance requirements for the current FOLR1 CDx assay, we systematically studied FOLR1 protein expression in a series of *n* = 51 NFTs obtained from women aged 26–83 years.

## Materials and methods

### Tissue specimens and patient age groups

This study included formalin‐fixed paraffin‐embedded (FFPE) tissue blocks from *n* = 51 consecutive salpingectomy specimens from the Hannover Medical School in Lower Saxony, Germany (Table 1). Salpingectomies were performed for different medical indications ranging from cesarean section with synchronous tubal sterilization to descensus uteri (Table 1). All specimens were fixed in neutral‐buffered formalin (4%) for 12–72 h. Each case was represented by one FFPE block harboring histologically confirmed NFT tissue. Median patient age was 46 years (range 26–83 years). Age is an established surrogate marker for estimating the menopausal status. Population‐based studies from Germany have shown that the median age at natural menopause is 50 years (range 45–56 years) [28, 29]. Accordingly, the collection of *n* = 51 NFTs was subdivided into three groups: premenopausal age (group A: <45 years), perimenopausal age (group B: 45–56 years), and postmenopausal age (group C: >56 years) (Table 1). In addition, this study also included a small series of *n* = 19 normal choroid plexus (CP) specimens and benign choroid plexus papillomas (CPPs) from mostly male patients (Table 2). All specimens were made anonymous for scientific purposes. This study was approved by the ethics committee of the Hannover Medical School (ID: 11482_BO_K_2024).

**Table 1:** Characteristics of NFT tissue samples

Group	A	B	C	Total
	(<45 years)	(45–56 years)	(>56 years)	
Number of cases	22	10	19	51
Age (years), median (min–max)	36 (26–44)	48 (45–55)	67 (57–83)	46 (26–83)
Salpingectomy context or medical indication				
Cesarean section and tubal sterilization	10 (45%)			10 (20%)
Tubal sterilization	6 (27%)	1 (10%)		7 (14%)
Ectopic pregnancy	2 (9%)			2 (4%)
Prophylactic salpingectomy	2 (9%)	7 (70%)	2 (11%)	11 (21%)
Endometriosis	1 (5%)	1 (10%)		2 (4%)
Benign epithelial ovarian neoplasia			8 (42%)	8 (15%)
Borderline/malignant epithelial ovarian neoplasia	1 (5%)			1 (2%)
Malignant neoplasia of the peritoneum			1 (5%)	1 (2%)
Germ cell tumor of the ovary			1 (5%)	1 (2%)
Malignant neoplasia of the cervix uteri		1 (10%)	1 (5%)	2 (4%)
Malignant neoplasia of the corpus uteri			3 (16%)	3 (6%)
Uterus myomatosus			2 (11%)	2 (4%)
Descensus uteri			1 (5%)	1 (2%)
Sum	22 (100%)	10 (100%)	19 (100%)	51 (100%)

**Table 2:** Characteristics of CP/CPP tissue samples

	A	B	C	Total
	<45 years	45–56 years	>56 years	
Number of cases	7	3	9	19
Age (years), median (min–max)	32 (3–42)	47 (47–52)	63 (57–85)	52 (3–85)
Gender				
Male	5 (71%)	2 (67%)	5 (56%)	12 (63%)
Female	2 (29%)	1 (33%)	4 (44%)	7 (37%)
Tissue type				
Normal choroid plexus	2 (29%)	1 (33%)	4 (44%)	7 (37%)
Choroid plexus papilloma	5 (71%)	2 (67%)	5 (56%)	12 (63%)

### FOLR1 immunohistochemistry

For IHC, 1 μm thick sections of FFPE tissue blocks were mounted on superfrost slides (Thermo Fisher Scientific, Rockford, IL, USA). Slides were deparaffinized and rehydrated conventionally and subjected to immunohistochemical staining using the FOLR1‐2.1 assay (Ventana) on a Benchmark Ultra automated stainer (Ventana). The CC1 standard program was used for antigen retrieval, and the Opti‐View kit (Ventana) for signal detection. The manufacturer (Ventana) installed and approved the correct FOLR1‐2.1 staining protocol by remote accession to the personal computer that operated the Benchmark Ultra automated stainer.

### Scoring of FOLR1 immunoreactivity

We used the standard histo‐score (H‐score) for evaluation of FOLR1 immunoreactivity [30]. The *H*‐score was calculated as *H* = (0 × *P*_0_) + (1 × *P*_1_) + (2 × *P*_2_) + (3 × *P*_3_), where *P*_*i*_ was the percentage of epithelial cells (estimated by eyeballing) stained at each staining intensity *i* (0, 1, 2, and 3). To accommodate differing staining intensities and distribution patterns of apical and basolateral FOLR1 expression, we implemented two separate H‐scores: an aH‐score for apical staining and a bH‐score for basolateral staining (each ranging from 0 to 300). For overall FOLR1 immunoreactivity of a given NFT specimen, we used a combined H‐score (cH‐score, ranging from 0 to 600), which was calculated as cH = aH + bH. Four pathologists (NS, MG, MR, and MC, termed observers herein) independently assessed aH‐ and bH‐scores for each of the *n* = 51 NFTs. Next, cH‐scores were calculated for each observer based on their individual aH‐ and bH‐scores (4 observers × 51 specimens = 204 cH‐scores). All observers were blinded to clinical parameters, such as age, as well as to the scoring results of the other observers. Further statistical evaluation was based on median aH‐, bH‐, and cH‐score per specimen (termed ‘consensus’ scores herein).

### Statistics

The intraclass correlation coefficient (ICC (3,1)) was used to assess agreement of aH‐, bH‐, and cH‐ scores obtained from different observers [31]. High versus low apical FOLR1 expression was defined according to the median aH‐score across the entire collection of *n* = 51 NFTs. High versus low basolateral FOLR1 expression was defined according to the median bH‐score. High versus low overall FOLR1 expression was defined according to the median cH‐score. Statistical significance of different FOLR1 expression among pre‐, peri‐, or postmenopausal age groups was assessed with the chi‐square test, the chi‐square test for trends, or Fisher's exact test using GraphPad Prism (version 5.00). Statistical significance of higher aH‐scores versus matched bH scores was determined with the Wilcoxon signed rank test. *P* values <0.050 were considered statistically significant.

## Results

### Histologic characterization of FOLR1 expression in NFTs

**Figure 1:**
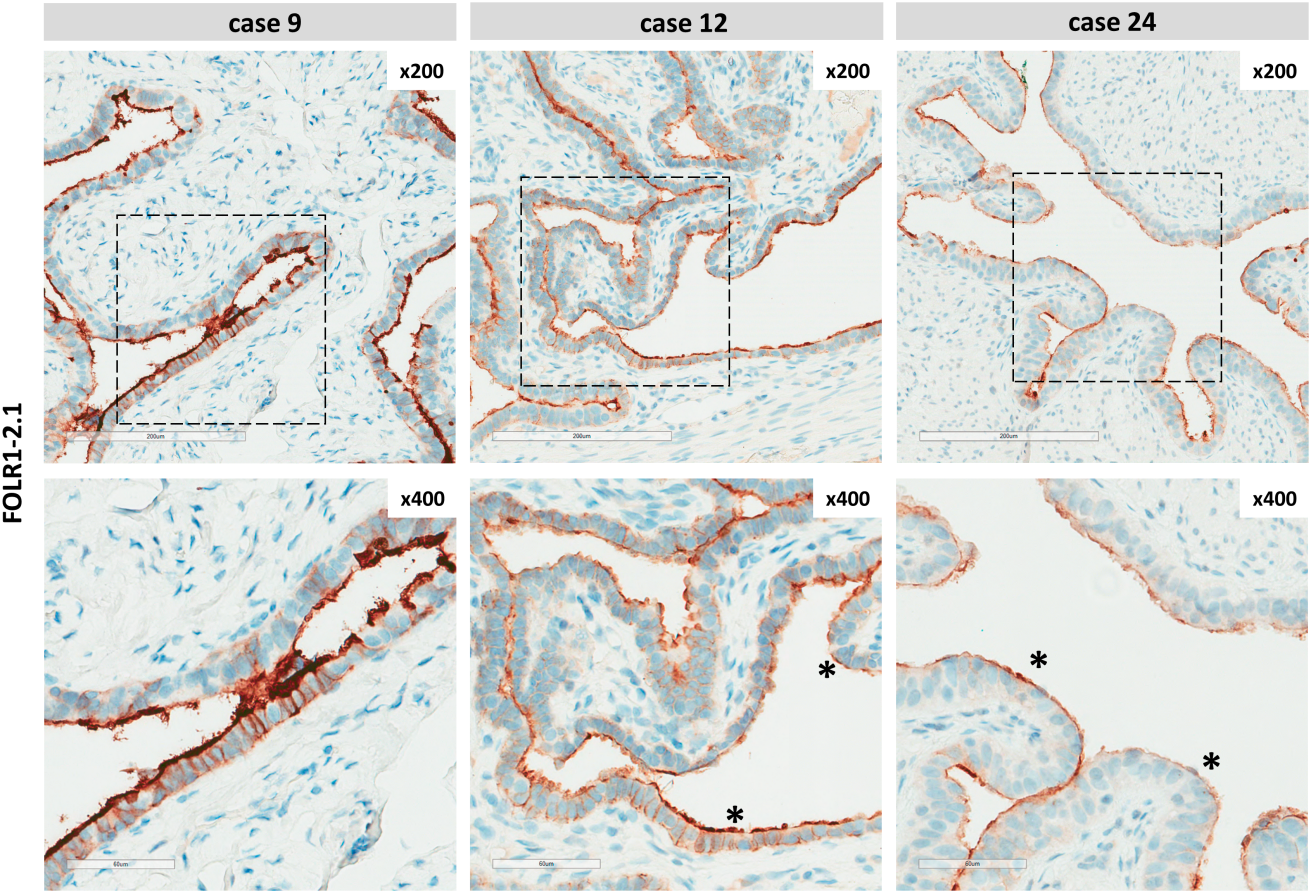
Variable FOLR1 protein expression in normal fallopian tubes (NFTs). Shown are representative IHC stainings for FOLR1 at ×200 magnification (top panels; scale bars correspond to 200 μm) and at ×400 magnification (bottom panels; scale bars correspond to 60 μm). Patient characteristics: case #9, 67‐year‐old female, under anti‐hormone therapy with tamoxifen for breast cancer; case #12, 31‐year‐old female, underwent cesarean section with synchronous tubal sterilization; case #24, 37‐year‐old female, underwent cesarean section synchronous with tubal sterilization. Asterisks highlight a subtle variation of the FOLR1 staining intensities along the mucosa within specimens.

Mesenchymal cells in the NFT stroma were FOLR1‐negative. The epithelial cells were FOLR1‐positive, but the staining intensity varied across the collection of cases (Figure 1). Apical cell membranes typically showed a somewhat stronger staining intensity than basolateral cell membranes. Some NFTs showed mostly strong FOLR1 immunoreactivity (3+) at apical membranes and moderate immunoreactivity (2+) at basolateral membranes (Figure 1, case 9). Other NFTs displayed mostly moderate staining intensity (2+) at apical membranes and weak staining intensity (1+) at basolateral membranes (Figure 1, case 12). A few NFTs showed weak staining intensity (1+) at apical membranes and lacked immunoreactivity at basolateral membranes (0) (Figure 1, case 24). In addition, NFTs often showed subtle, patchy variations of FOLR1 staining intensities along the mucosa within individual specimens (Figure 1, asterisks).

### Variable FOLR1 immunoreactivity scores in NFTs

**Figure 2:**
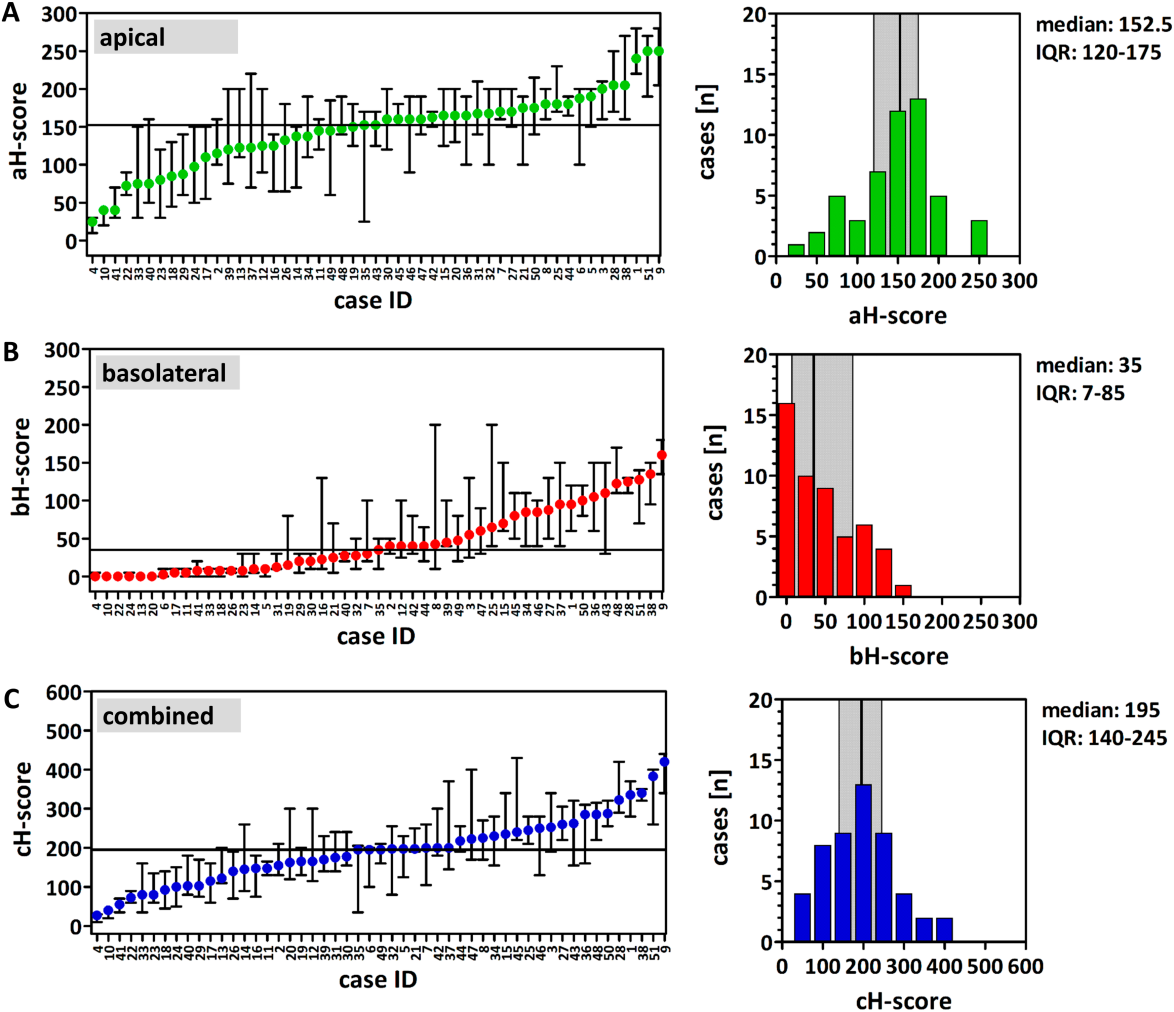
Assessment of FOLR1 expression in *n* = 51 normal fallopian tubes (NFTs) using H‐scores. (A) Apical staining (aH‐scores, green color). (B) Basolateral staining (bH‐score, red color). (C) Overall staining (cH‐score, blue color). Panels on the left side present waterfall plots showing median (‘consensus’) aH‐, bH‐, and cH‐scores across the entire collection. Cases are ordered according to increasing immunoreactivity. Specimen IDs are plotted on the *x*‐axis. Immunoreactivity scores are plotted on the *y*‐axis. Dots represent median (‘consensus’) scores for each individual specimen, obtained through independent evaluation by four observers. Whiskers indicate minimal/maximal scores out of the four observers. Horizontal lines indicate the median aH‐, bH‐, and cH‐score across the entire collection (152.5, 35, and 195, respectively). Panels on the right side illustrate the distribution of aH‐, bH‐ and cH‐scores in histograms. Vertical lines highlight the median, and the gray margins indicate the interquartile range (IQR). Case IDs of the ten NFTs obtained from women who underwent cesarean section with synchronous tubal sterilization were as follows: case #10, #11, #12, #13, #14, #21, #22, #24, #25, and #26. Case IDs of the two NFTs obtained from women who underwent salpingectomy for ectopic pregnancy were case #18 and case #20.

Four pathologists independently assessed FOLR1 aH‐ and bH‐scores for each of the n = 51 NFT specimens. Their corresponding cH‐scores were calculated as described in the methods section. H‐scores obtained by different observers showed moderate to good agreement (supplementary material, Figure S1A–D). Further statistical evaluation was based on median (‘consensus’) aH‐, bH‐, and cH‐score per specimen (Figure 2). The median aH‐score across the entire collection of NFTs was 152.5 [interquartile range (IQR): 120–175] (Figure 2A). The median bH‐score across all NFTs was 35 (IQR: 7–85) (Figure 2B). The median cH‐score of all NFTs was 195 (IQR 140–245) (Figure 2C). Apical immunoreactivity scored higher than basolateral immunoreactivity (median aH‐score: 152.5, median bH‐score: 35, *p* < 0.001) (supplementary material, Figure S1E). Taken together, NFTs showed variable FOLR1 immunoreactivity scores.

### Low FOLR1 immunoreactivity is associated with premenopausal age

**Figure 3:**
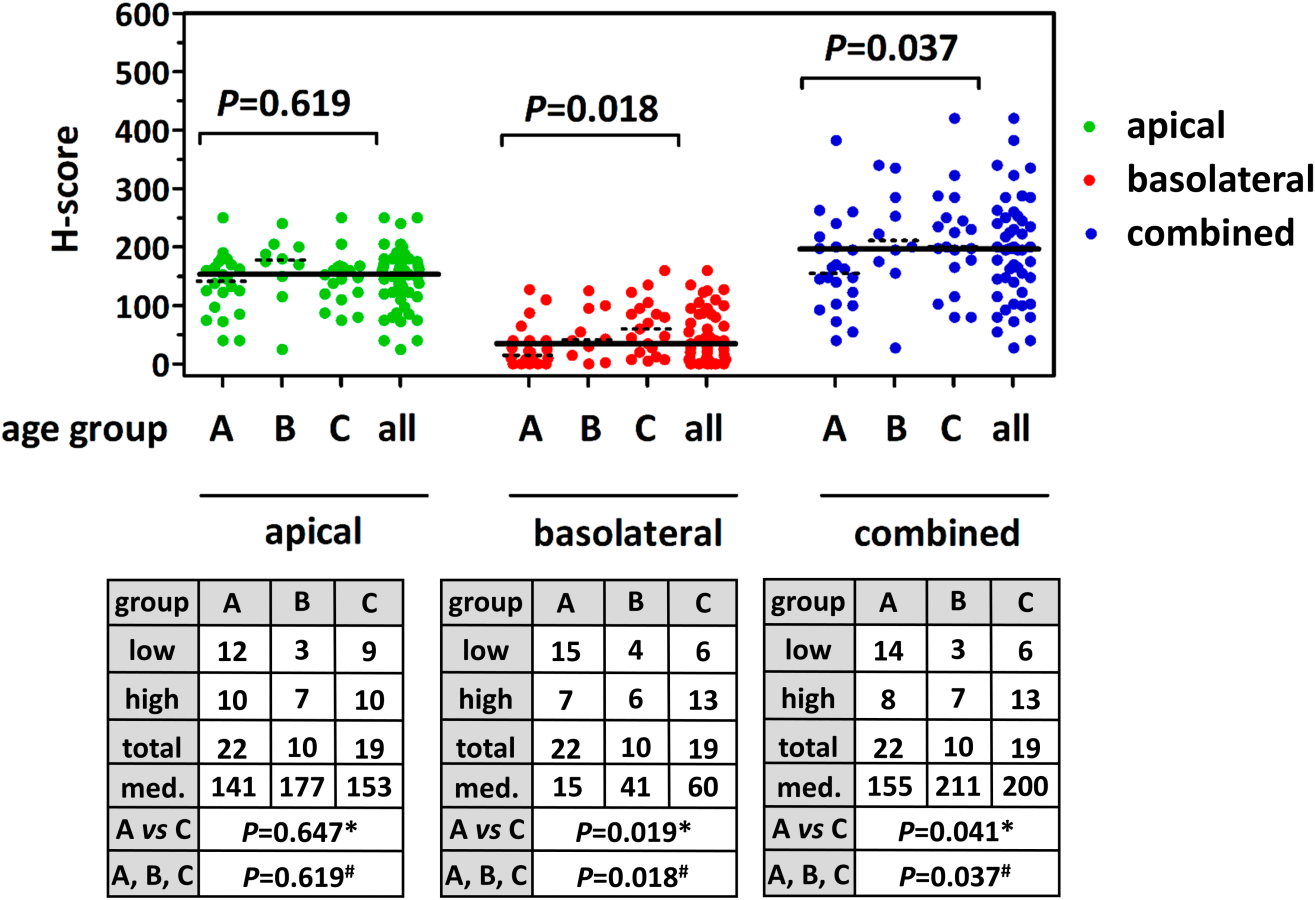
Low FOLR1 immunoreactivity in normal fallopian tubes (NFTs) is associated with premenopausal age. The top panel shows scatter plots with median (‘consensus’) aH‐scores (green), bH‐scores (red), and cH‐scores (blue) for all NFTs (*n* = 51). Broad horizontal lines indicate the median aH‐score (152.5), the median bH‐score (35), and the median cH‐score (195) across the entire collection. Dotted lines indicate median aH‐, bH, and cH‐scores in different age groups. Age groups were as follows: A, <45 years; B, 45–56 years; C, >56 years (corresponding to premenopausal, perimenopausal, and postmenopausal age). The bottom panels show the results in tables. High versus low FOLR1 expression was defined as <median versus ≥median aH‐, bH‐, or cH‐score. Statistical significance was evaluated using contingency tables and the *chi‐square test (group A versus C), or the ^#^chi‐square test for trends (groups A, B, and C).

NFT specimens were assigned to three age groups corresponding to premenopausal age (group A: <45 years), perimenopausal age (group B: 45–56 years), and postmenopausal age (group C: >56 years) (Table 1). Low overall FOLR1 expression (cH‐score <195) was associated with premenopausal age (*p* = 0.037) (Figure 3). Apical immunoreactivity was age‐independent (*p* = 0.619), but low or absent basolateral immunoreactivity (bH‐score <35) was also associated with premenopausal age (*p* = 0.018). Taken together, NFTs showed an age‐dependent FOLR1 expression pattern.

### Low FOLR1 immunoreactivity in NFTs from pregnant women

**Figure 4:**
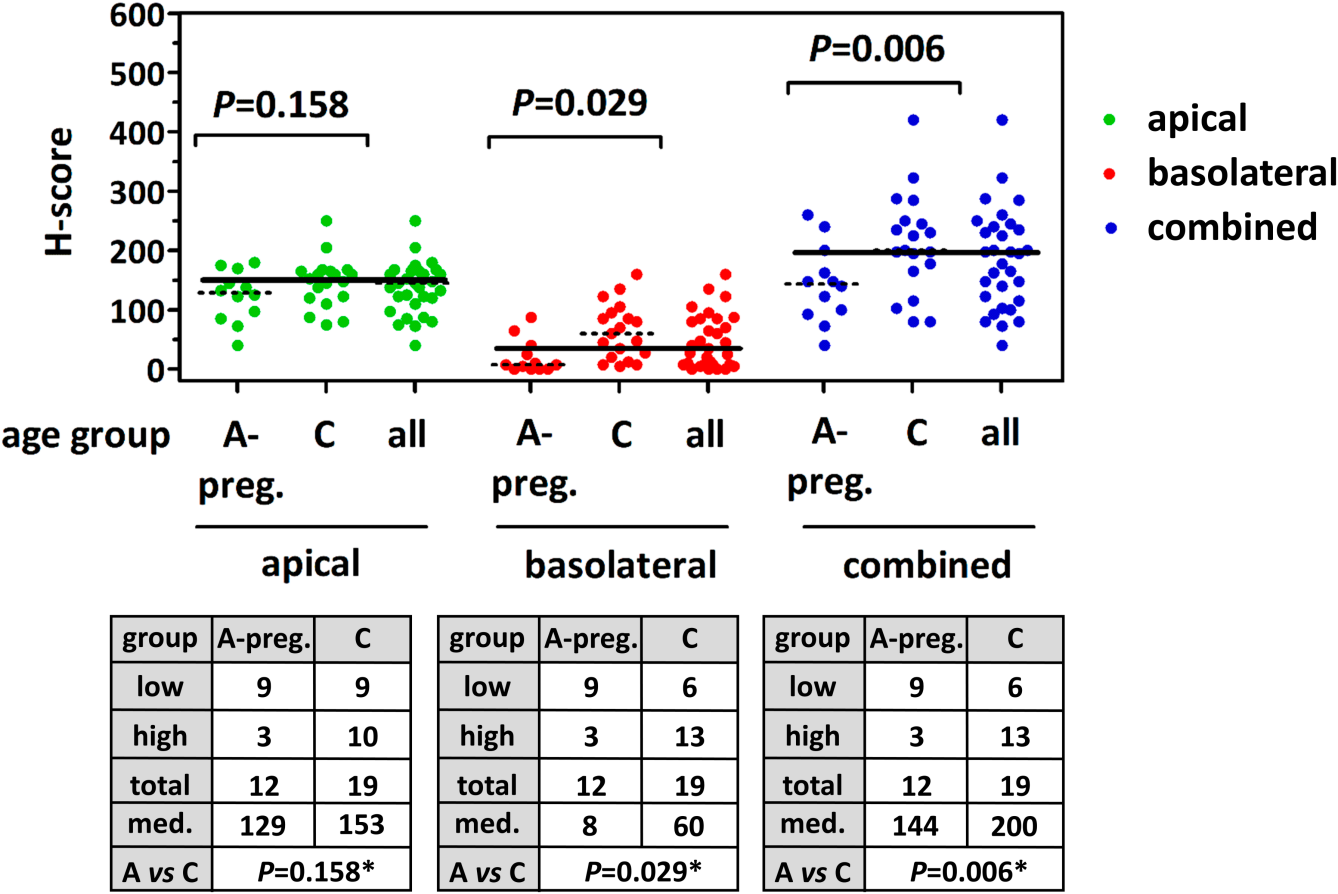
Low FOLR1 immunoreactivity in normal fallopian tubes (NFTs) from pregnant women. The top panel shows scatter plots with median (‘consensus’) aH‐scores (green), bH‐scores (red), and cH‐scores (blue) for NFTs in the refined data set (*n* = 31). This included NFTs from premenopausal, pregnant women (group A‐preg., *n* = 12) and NFTs from women at postmenopausal age (group C, *n* = 19). Broad horizontal lines indicate the median aH‐score (152.5), the median bH‐score (35), and the median cH‐score (195) across the entire collection of NFTs (*n* = 51). Dotted lines indicate median aH‐, bH, and cH‐scores in different age groups. The bottom panels show the results in tables. High versus low FOLR1 expression was defined as <median versus ≥median aH‐, bH‐, or cH‐score. *Statistical significance was evaluated using contingency tables and Fisher's exact test (group A‐preg. versus C).

Five out of six (83%) NFTs with absence of basolateral FOLR1 expression (bH‐score 0) were obtained from pregnant women aged ≤37 years, who underwent cesarean section with synchronous tubal sterilization (cases #10, #22, #24, and #13) or salpingectomy for ectopic pregnancy (case #20) (Figure 2B). This prompted us to perform additional statistical analyses in a refined subset of *n* = 31 NFTs, including only tissue specimens obtained from pregnant women (*n* = 12) and from women at postmenopausal age (*n* = 19). Low/absent basolateral FOLR1 expression (bH‐score <35) and low overall FOLR1 expression (cH‐score <195) were both associated with NFTs obtained from pregnant women (*p* = 0.029 and *p* = 0.006) (Figure 4). The highest aH‐, bH‐, and cH‐scores of the entire collection were observed in a 67‐year‐old postmenopausal female, who was treated with anti‐hormone therapy (tamoxifen) for luminal breast cancer diagnosed 1 year earlier (case #9) (Figure 2A–C). Taken together, hormonal factors, including pregnancy, influence FOLR1 expression in NFTs.

### FOLR1 expression in choroid plexus

**Figure 5:**
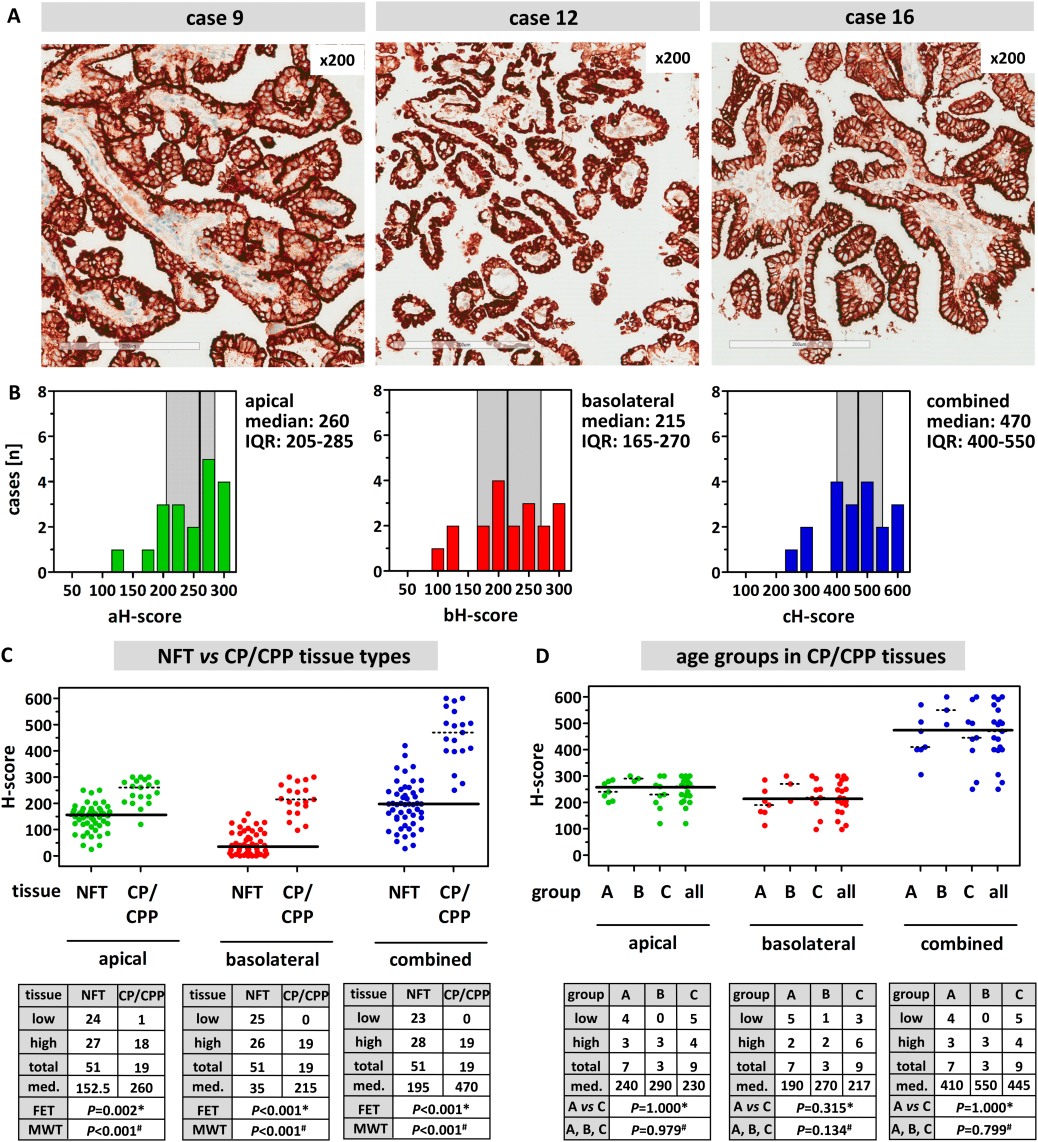
FOLR1 expression in choroid plexus and choroid plexus papilloma (CP/CPP) specimens. (A) Shown are representative IHC stainings for FOLR1 at ×200 magnification. Scale bars correspond to 200 μm. Patient characteristics: case #9, CPP from a 60‐year‐old male patient; case #12, CPP from a 52‐year‐old male patient; case #16, CPP from a 3‐year‐old male patient. (B) Distribution of median (‘consensus’) aH‐scores (green), bH‐scores (red), and cH‐scores (blue) across the entire series of CP/CPP specimens. (C) Comparison of FOLR1 immunoreactivity in NFT and CP/CPP specimens. The top panel shows scatter plots with median (‘consensus’) aH‐, bH‐, and cH‐ for all NFT specimens (*n* = 51) and for all CP/CPP specimens (*n* = 19). Broad horizontal lines indicate the median aH‐score (152.5), the median bH‐score (35), and the median cH‐score (195) across the entire series of NFT specimens. The dotted lines indicate median aH‐, bH, and cH‐score across CP/CPP specimens. The bottom panels show the results in tables. High versus low FOLR1 expression was defined as <median versus ≥median aH‐, bH‐, or cH‐scores derived from NFT specimens. Med.; median. Statistical significance was evaluated using contingency tables and *Fisher's exact test (group A versus C), or the ^#^Mann–Whitney test. (D) Relationship between FOLR1 immunoreactivity and age groups in CP/CPP specimens. The top panel shows scatter plots with median (‘consensus’) aH‐, bH‐, and cH‐scores for all CP/CPP specimens. Broad horizontal lines indicate the median aH‐score (260), the median bH‐score (215), and the median cH‐score (470) across the entire series of CP/CPP specimens. The dotted lines indicate median aH‐, bH, and cH‐scores in different age groups. Age groups were as follows: A, <45 years; B, 45–56 years; C, >56 years. The bottom panels show the results in tables. High versus low FOLR1 expression was defined as <median versus ≥median aH‐, bH‐, or cH‐score. Med.; median within each age group. Statistical significance was evaluated using contingency tables and *Fisher's exact test (group A versus C), or the ^#^chi‐square test for trends (groups A, B, and C).

Choroid plexus (CP) epithelial cells have been reported to express the highest levels of FOLR1 protein of all human tissue types [4, 6]. CP may offer an option for an additional OPC. Hence, we also assessed FOLR1 immunoreactivity in a small series of *n* = 19 CP specimens or choroid plexus papillomas (CPPs) from mostly male patients (Table 2). CP/CPP specimens showed higher FOLR1 immunoreactivity scores than NFTs, especially at basolateral cell membranes (Figure 5A–C). The median aH‐score across the entire series of CP/CPPs was 260 (IQR: 205–285). The median bH‐score was 215 (IQR: 165–270). The median cH‐score was 470 (IQR 400–550) (Figure 5B). There was no association between age group and FOLR1 immunoreactivity in CP/CPP specimens (Figure 5D).

## Discussion

Eligibility of ovarian carcinoma patients for MIRV‐S therapy is determined by IHC using an FDA‐approved CDx assay (FOLR1‐2.1 assay, Ventana) [12]. This assay classifies ovarian carcinomas as FOLR1‐positive if ≥75% of viable tumor cells show FOLR1 immunoreactivity with at least 2+ staining intensity [1, 11, 12, 32]. The FOLR1‐2.1 CDx assay requires preparation of NFTs as an external OPC for quality assurance, assay performance monitoring, and to aid in the interpretation of different staining intensity (0, 1+, 2+, or 3+) [12]. According to the manufacturer's instructions, NFT epithelial cells provide a reference for strong staining intensity (3+) at apical cell membranes, a reference for moderate staining intensity (2+) at basolateral cell membranes, and a reference for absent staining (0) in the mesenchymal stroma [12]. According to the current manufacturer's instructions, evaluation of any FOLR1‐stained tumor tissue must be rejected if the external OPC prepared from NFT tissue lacks FOLR1 immunoreactivity at basolateral cell membranes or shows only weak FOLR1 staining intensity at basolateral cell membranes. Our laboratory introduced the FOLR1‐2.1 assay in December 2024. In early 2025, lot‐to‐lot‐validation showed that some NFTs displayed only weak staining (1+) or absent staining (0) at basolateral membranes, and the respective NFTs were excluded from OPC use. FOLR1 has been known as a target of ER‐dependent gene repression for more than 20 years [25]. We hypothesized that the variable FOLR1 staining characteristics in NFTs are related to the menopause status. To test this hypothesis, we systematically studied FOLR1 immunoreactivity in a collection of NFTs. Our analysis revealed reduced FOLR1 expression in NFTs from women at premenopausal age. This is consistent with the known repression of FOLR1 by ER‐signaling [25]. Most importantly, we noted that NFTs obtained from pregnant women who underwent cesarean section with synchronous tubal sterilization often showed a complete absence of FOLR1 immunoreactivity at basolateral cell membranes. According to the manufacturer of the FOLR1‐2.1 CDx assay, this staining pattern indicates technically invalid staining. However, our findings suggest that such a pattern is normal for NFTs from pregnant women, likely reflecting hormonal repression of FOLR1 in the fallopian tube mucosa.

This finding is relevant for patient care. Most institutions that offer FOLR1 assessment for ovarian carcinoma patients probably derive their OPCs from NFTs obtained from tubal sterilization performed synchronously with cesarean sections. In fact, this is presumably the most common medical constellation through which tissue archives of pathology institutions acquire NFTs from essentially healthy individuals. Absent, reduced, or faint (1+) basolateral FOLR1 immunoreactivity in OPCs prepared from NFTs of premenopausal and/or pregnant women possibly bears the risk of misidentifying staining intensities in the tumor tissue, if reading the slide is not rejected based on the unacceptable OPC. Misidentifying different FOLR1 staining intensities could unintentionally inflate the proportion of FOLR1‐positive ovarian carcinoma patients and might thereby even impact on the proportion of patients that derive benefit from MIRV‐S therapy. Reduced FOLR1 expression in NFTs from premenopausal and/or pregnant women could have been described earlier. The developers of the FOLR1 2.1 assay paid great attention to assay reproducibility [12]. However, a systematic analysis of FOLR1 expression in the selected positive control tissue type was not reported [12]. Expert recommendations have underscored the importance of the iCAPCs concept for the development of new assays [13, 14, 15]. For FOLR1, it seems that evidence‐based knowledge about different expression patterns in NFTs was insufficient at the time of assay development.

Recently, Scheiter *et al* reported the results of the first German round robin test for FOLR1 assessment in ovarian carcinoma [33]. More than 75% of laboratories using alternative FOLR1‐assays (non‐FOLR1‐2.1) failed to pass the round robin test [33]. Among laboratories that implemented the original FOLR1‐2.1 CDx assay, the failure rate was still 17% of participating laboratories [33]. Problems related to the interpretation of staining (intensities) were mentioned as the most common cause for unsuccessful participation. It is tempting to speculate whether or not unacceptable or borderline acceptable OPCs could have played a role in the mediocre results in this first round robin test [33]. However, OPCs were not systematically evaluated in this round robin test. Synthetic calibrators, like those recently developed for PD‐L1 and HER2 epitopes [18, 20, 21], would be a promising tool for the evaluation of FOLR1 IHC assay performance in different laboratories.

The limitations of the present study include the fact that estrogen serum levels and anamnestic menopausal status data were not available. This is due to the retrospective, exploratory approach of the present study (Table 1). Nonetheless, the results of this study are reliable because age is a well‐established surrogate marker for menopausal status [28, 29]. FOLR1 expression characteristics in premenopausal women may even vary depending on the phase of the menstrual cycle. However, this is beyond the scope of the present study.

For now, it is important to communicate that CP/CPP tissue qualifies as an additional OPC for FOLR1. CP/CPP tissue shows stronger immunoreactivity for FOLR1 than NFTs. Moreover, CP/CPP tissue does not show relevant age‐dependent variation of FOLR1 immunoreactivity. Currently, our custom‐made OPCs/ONCs for the FOLR1 CDx assay are mini‐tissue microarrays (mini‐TMAs) that include choroid plexus (FOLR1‐positive, mostly 3+ staining intensity), spleen (FOLR1‐negative, staining intensity 0), and postmenopausal NFT tissue (FOLR1‐positive, variable intensity and staining pattern, 1+ to 2+ at basolateral membranes for demonstration of LLOD, 2+ to 3+ at apical membranes). To our knowledge, this mini‐TMA design effectively describes the first clinically validated iCAPCs for the FOLR1 assay.

The conclusions of this study are simple: (1) OPCs for predictive IH markers or class II assays must be validated through both (a) initial validation studies, such as presented in this work, and (b) lot‐to‐lot validation protocols for all subsequent OPC preparations. (2) NFT tissue from postmenopausal women is appropriate for OPCs for FOLR1 IHC and meets the performance requirements for the current CDx assay.

## Author contributions statement

MC designed this study. LDK, PH and HC managed the FFPE block collection and carried out IHC stainings. MC, MR, NS and MG scored the IHC stainings. LDK, AN and MC performed statistical analyses. JH, EK and CH contributed tissue specimens and/or clinicopathological data. All authors contributed to data collection, data analysis and interpretation. AN and MC wrote the manuscript. All authors reviewed and approved the final manuscript version.

## Supporting information


**Figure S1.** Inter‐observer agreement of FOLR1 H‐scores.

## Data Availability

All raw data are available upon reasonable request.
