# Supplementary material for: Reduced folate receptor alpha (FOLR1) protein expression in fallopian tubes from premenopausal women: implications for the FOLR1 CDx assay for mirvetuximab‐soravtansine therapy
Source: J Pathol Clin Res. 2026 Apr 23;12(3):e70091. doi: 10.1002/2056-4538.70091 (PMC13103951; doi:10.1002/2056-4538.70091)
Supplement: Supplementary file 1 — Figure S1. Inter‐observer agreement of FOLR1 H‐scores. [file CJP2-12-e70091-s001.pdf]

Reduced folate receptor alpha (FOLR1) protein expression in fallopian tubes from premenopausal women: implications for the FOLR1 CDx assay for mirvetuximab-soravtansine therapy

A Nasdala *et al*. *J Pathol Clin Res* https://doi.org/10.1002/2056-4538.70091

Supplementary Figure S1

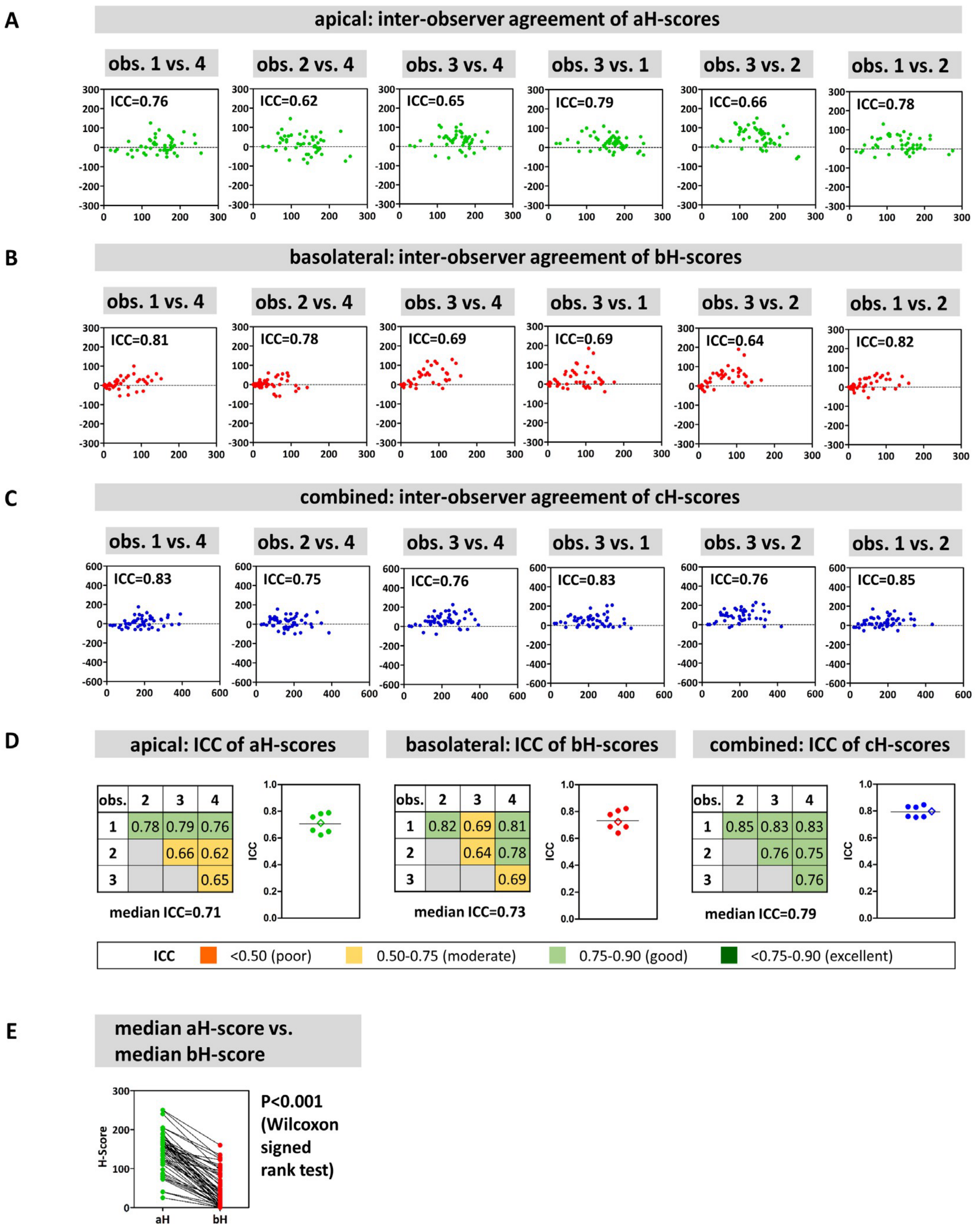

Figure S1. Inter-observer agreement of FOLR1 H-scores in normal fallopian tubes (NFTs). Four pathologists [observer (obs.) 1 to 4] independently assessed aH-, and bH-scores in *n* = 51 NFTs and the corresponding cH-scores were calculated based on their individual aH-, and bH-scores. (A) Pairwise Bland-Altman plots for aH-scores. From left to right, each diagram represents a different pair of observers. Each dot represents a NFT specimen. For each specimen, the average aH-score of the two observers is plotted on the *x*-axis. The difference between the aH-scores by the two observers is plotted on the *y*-axis. Pairwise intraclass correlation coefficients (ICC) are shown in the upper left corners of the diagrams. (B) Pairwise Bland-Altman plots for bH-scores. (C) Pairwise Bland-Altman plots for cH-scores. (D) Median ICCs for aH-, bH-, and cH-scores. (E) Comparison of median ('consensus') aH-, and bH-scores. Consensus aH-scores were always higher than matched consensus bH-scores. Statistical significance was determined with the Wilcoxon signed rank test.
